# Health perception and physical activity level profile of bus drivers of urban public transportation in Chapecó-SC

**DOI:** 10.47626/1679-4435-2022-958

**Published:** 2024-08-05

**Authors:** Moacir Roque Fernandes, Dayanne Sampaio Antonio, Rafael Cunha Laux

**Affiliations:** 1 Educação Física, Universidade do Oeste de Santa Catarina, Chapecó, SC, Brazil; 2 Educação Física, Universidade Federal do Paraná, Curitiba, PR, Brazil

**Keywords:** bus driver, health, exercise, quality of life, motorista, saúde, atividade física, qualidade de vida

## Abstract

**Introduction:**

Urban public transportation workers are of great social importance in cities. Working conditions can negatively affect the workers’ health, causing illness and loss of quality of life.

**Objectives:**

To analyze the perception of health and level of physical activity of bus drivers in urban public transportation in Chapecó, state of Santa Catarina.

**Methods:**

This study was characterized as descriptive, cross-sectional and field. It was carried out with 46 bus drivers, both genders, aged between 21 and 59 years. An anamnesis form was applied to characterize the participants and to assess the level of physical activity, the reduced International Physical Activity Questionnaire version VIII was used. The research took place in August and September 2021. Data analysis was performed descriptively, using means, standard deviation, relative and absolute frequency.

**Results:**

There was a prevalence of 91.30% of practitioners of physical activity The perception of health considered good was 71.74%, being associated with the absence of chronic diseases. The predominant level of physical activity was physically active (63.05%).

**Conclusions:**

The vast majority of bus drivers presented a good perception of health, practiced physical activity, considered their work activities to be good and mentioned not taking medication for the treatment of chronic diseases.

## Abstract (pt)

**Introdução:**

Os trabalhadores do transporte coletivo urbano têm grande importância social nas cidades. Todavia, as condições de trabalho podem afetar negativamente a saúde do trabalhador, causando adoecimento e perda de qualidade de vida.

**Objetivos:**

Analisar a percepção de saúde e o nível de atividade física de motoristas de ônibus do transporte coletivo urbano de Chapecó, no estado de Santa Catarina.

**Métodos:**

Este estudo caracterizou-se como descritivo, transversal e de campo. Foi realizado com 46 motoristas de ônibus, de ambos os sexos, com idade entre 21 e 59 anos. Aplicou-se uma ficha de anamnese para caracterização dos participantes, e, para avaliar o nível de atividade física, utilizou-se o International Physical Activity Questionnaire, versão VIII reduzida. A pesquisa ocorreu nos meses de agosto e setembro de 2021. A análise de dados foi realizada de maneira descritiva, utilizando média, desvio padrão e frequências relativa e absoluta.

**Resultados:**

Evidenciou-se prevalência de 91,30% de praticantes de atividade física. A percepção de saúde considerada boa foi de 71,74%, sendo associada à ausência de doenças crônicas. O nível de atividade física predominante foi de “ativo fisicamente” (63,05%).

**Conclusões:**

Os motoristas apresentam, na sua grande maioria, uma boa percepção de saúde, são praticantes de atividade física, consideram boas as suas atividades de trabalho e mencionam não tomar medicamentos para tratamento de doenças crônicas.

## INTRODUCTION

Public transport has become increasingly important socially and economically as urbanization grows continuously, transporting millions of passengers who depend on it to meet their basic needs.^1^ It is of significant importance that operators behave in a way that enables them to perform this essential service to the population. Some poorly executed actions in this work can endanger not only the drivers’ lives, but also the lives of many people.^2^

The transportation of passengers accounts for around 70% of motorized transport in Brazilian cities, and buses are almost exclusively responsible for commuting.^3^ Workers in this industry have both a macro-and a micro-workplace, namely the traffic and the bus. Given these particular features, no other professional is subjected to the pressures of the road environment as much as bus drivers.^4^

In addition, bus drivers are bound to an unhealthy working routine, with long working hours, requiring constant attention, irregular meal times, the risk of accidents, sedentary lifestyles, repetitive movements, altered vision, emotional stress, among others.^5,6^ Therefore, drivers’ lifestyle and working conditions have been a cause for concern among employers because, in addition to factors within the organization, they need to think about their health, that is, their diet, exercise, and well-being, as these are determinants of their quality of life inside and outside their workplace.^5,7^

However, advances in technology have allowed people to have easier access to machines and material goods, placing exercise on a secondary level, in addition to a variety of eating habits with a higher intake of saturated fats and sugars.^8^ These conditions therefore lead to the emergence of chronic noncommunicable diseases such as hypercholesterolemia, type-2 diabetes mellitus, obesity, cardiovascular diseases, and hypertension.^9^ This raises questions about the quality of life of bus drivers, yet only a few studies are referenced in the literature, especially when it comes to the health and lifestyle of this group of professionals.^10^ For these reasons, this study aimed to analyze the perception of health and the level of exercise of urban public transport bus drivers in the municipality of Chapecó, SC, Brazil.

## METHODS

This is a descriptive, cross-sectional, field study. The Ethics Research Committee of the Universidade do Oeste de Santa Catarina approved this study according to opinion No. 4.939.428/2021 and Certificate of Submission for Ethical Appreciation No. 49248921.0.0000.5367.

This study complied with all guidelines in National Health Council Resolution No. 466/12, which requires participants to have signed a free and informed consent form (ICF).

**Figure 1:**
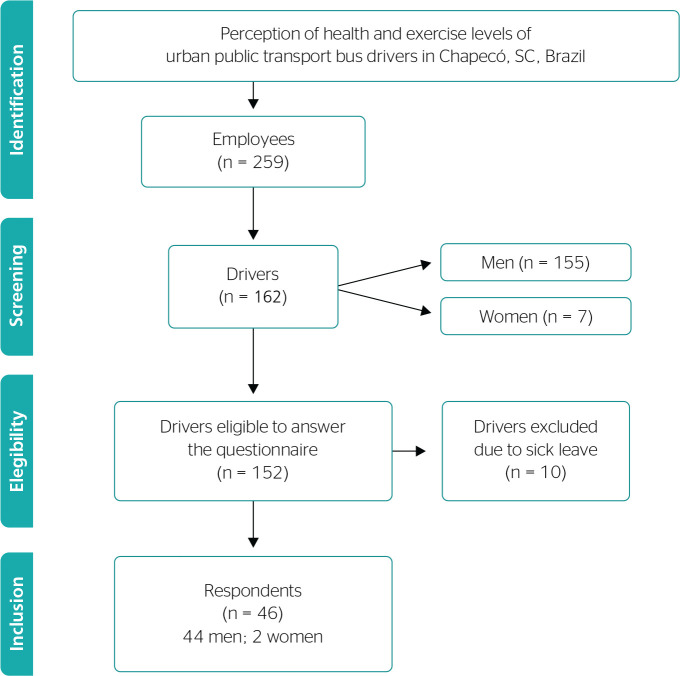
Flowchart with eligibility criteria.

This study included 46 bus drivers of either sex, between 21 and 59 years old, out of 161 bus drivers from an urban public transport company in Chapecó (Figure 1). These drivers filled in an anamnesis form to assess their overall health and rest conditions.

The International Physical Activity Questionnaire (IPAQ) short form 8, validated by Matsudo et al.^11^ was used to assess the level of exercise. It includes 5 classification criteria on the level of exercise, namely sedentary, insufficiently active A, insufficiently active B, active, and very active.^12^

Prior to data collection, a video introducing the investigators was sent to the study group to explain the objectives and justify the importance of carrying out this study. The anamnesis and IPAQ were then applied using a Google Forms^®^ form, which was provided through the company’s social networks for communication with their employees, or printed out for each participant. The investigators contacted the study group through a social network (WhatsApp^®^), which was administered by the company’s human resources department. The questionnaire was collected during August and September 2021.

The data were analyzed descriptively, using mean, standard deviation (SD), and relative and absolute frequencies. All data were analyzed using Excel^®^ for Windows, version 365.

## RESULTS

The study included 46 urban public transport bus drivers in the city of Chapecó, SC, Brazil. The average age was 37.1 years (SD 10.0), and the mean time working as a bus driver was 89.2 months (SD 107.1). The drivers’ perception of their exercise levels was 91.30%, who reported having exercise routines, as shown in Table 1.

**Table 1:** Profile of public transport bus drivers in Chapecó, SC, Brazil

	Overall group (np = 46)	Women (np = 2)	Men (np = 44)
	M(SD)	M(SD)	M(SD)
Age (years)	37.1 (10.0)	37.8 (12.4)	37.1 (10.0)
Time spent daily on online media (minutes)	214.4 (147.2)	180.0 (0.0)	216.0 (150.4)
Length of time at work (months)	89.2 (1071)	111.5 (130.8)	88.1 (107.6)
Length of daily working time (minutes)	462.1 (36.6)	440.0 (0.0)	463.1 (371)
	**n (%)**	**n (%)**	**n (%)**
Sex			
Women	2(4.30)		
Men	44 (95.70)		
Exercise			
Yes	42 (91.30)	2 (100.00)	40 (90.91)
No	4 (8.70)	0 (0.00)	4 (9.09)

SD = standard deviation; M = mean; n = absolute frequency; np = number of participants.

Table 2 shows the results of the drivers’ quality of life, showing that 91.30% of the participants had no chronic illnesses, their current health perception was considered good (71.74%) and their stress level was considered moderate (67.39%).

**Table 2:** Quality of life of urban transport bus drivers in Chapecó, SC, Brazil

	Overall group (np = 46)	Women (np = 2)	Men (np = 44)
	M(SD)	M(SD)	M(SD)
Daily sleeping time (minutes)	402.06 (73.2)	390.00 (42.4)	403.20 (74.5)
	**n (%)**	**n (%)**	**n (%)**
Sound sleeping habits			
Yes	35 (76.09)	2(100.00)	33 (75.00)
No	11 (23.91)	0 (00.00)	11 (25.00)
Chronic illnesses			
Yes	4 (8.70)	0 (0.00)	4 (9.09)
No	42 (91.30)	2(100.00)	40 (90.91)
How would you rate your current health status?			
Fair	5 (10.87)	0 (0.00)	18(4091)
Good	33(71.74)	2(100.00)	18(4091)
Great	8(17.39)	0 (0.00)	8(18.18)
How often do you think you sleep well?			
Often	18(39.13)	0 (0.00)	18(4091)
Usually	20 (43.48)	2(100.00)	18(4091)
Always	8(17.39)	0 (0.00)	8(18.18)
How would you rate your stress levels?			
Poor	3 (6.52)	0 (0.00)	3(6.82)
Fair	31 (67.39)	2(100.00)	29 (65.91)
Good	10 (21.74)	0 (0.00)	10 (22.73)
Great	2(4.35)	0 (0.00)	2 (4.55)
How would you rate your work activities?			
Fair	5 (10.87)	0 (0.00)	5(11.36)
Good	35 (76.09)	1 (50.00)	34 (77.27)
Great	6 (13.04)	1 (50.00)	5(11.36)

SD = standard deviation; M = mean; n = absolute frequency; np = number of participants.

Table 3 shows the results of exercise levels and sedentary behavior, which 36.96% of participants were considered as active. This result is similar to stratification per sex, which shows that 50.00% of women and 36.36% of men are considered active.

**Table 3:** Data on the exercise levels and sedentary behavior of urban transport bus drivers in Chapecó, SC, Brazil

	Overall group (np = 46)	Women (np = 2)	Men (np = 44)
	n (%)	n (%)	n (%)
Exercise levels Highly active	12 (26.09)	1 (50.00)	11 (25.00)
Active	17 (36.96)	1 (50.00)	16 (36.36)
Insufficiently active A	9 (19.57)	0 (0.00)	9 (20.45)
Insufficiently active B	4 (8.70)	0 (0.00)	4 (9.09)
Sedentary	4 (8.70)	0 (0.00)	4 (9.09)
	M (DP)	M (DP)	M (DP)
Sedentary behavior Daily sitting time on week days (minutes)	473.7 (175.0)	430.0 (14.1)	475.7 (178.8)
Daily sitting time on the weekend (minutes)	347.6 (161.7)	420.0 (254.6)	344.3 (160.0)
Overall sitting time (minutes)	821.3 (274.7)	850.0 (268.7)	820.0 (278.0)

SD = standard deviation; M = mean; n = absolute frequency; np = number of participants.

## DISCUSSION

This study analyzed the perception of health and exercise levels of urban public transport bus drivers in the municipality of Chapecó, SC, Brazil, and found that the participants had a good perception of their health and work activities, had a moderate level of stress, had no chronic diseases, and were considered physically active.

Similarly, Garbaccio & Pereira^13^ assessed the self-perception of health of urban bus drivers and corroborated the findings of this study concerning chronic diseases, finding that of the 324 bus drivers analyzed, 80.3% reported having no chronic diseases. In addition, 72.8% of these drivers defined their health as good, thus similar to our findings.

In this respect, Prado^14^ found that 55% of 322 drivers from Aracajú, SE, Brazil, were also happy with their health. Equally, Moura-Neto & Silva^15^ found that 54.1% of 225 drivers assessed in Pelotas, RS, Brazil, reported good health.

A total of 76.09% of drivers rated their work activities as good. It corroborated the findings of Moura-Neto & Silva,^15^ who investigated the diagnosis of working conditions, health, and lifestyle indicators of public transport workers in Pelotas, RS, Brazil, which 92.80% of drivers responded positively when asked if they were happy with their jobs. Compared to the findings of this study, Garbaccio & Pereira^13^ found that 95.10% of drivers were happy with their jobs and were satisfied with all aspects investigated (management; their jobs; colleagues; passengers).

The data obtained in this study showed that 67.39% of drivers reported a moderate level of stress symptoms. Thus, urban bus drivers are among the most stressful occupations in Brazil, due to a number of traffic-related and customer support issues.^16^

Almeida^17^ evaluated 20 private drivers, 20 taxi drivers, and 20 bus drivers in Recife, PE, Brazil, and found that 36.90% of bus drivers had the highest stress levels. Similarly, Tavares^18^ found that 34.3% of the 134 drivers in Uberlândia, MG, Brazil, had symptoms of stress. Finally, of the 322 bus drivers studied in Aracajú, SE, Brazil, 46.89% showed some level of stress.^19^

As for the level of exercise, this study found that 36.96% of respondents reported to be physically active. However, Prado^14^ found that 77% of the drivers assessed did not meet the recommended levels of exercise. Garbaccio & Pereira^13^ also found that 50.60% of the 324 drivers were completely sedentary on weekdays.

This can be explained on the grounds that there is a negative correlation between exercise and stress, indicating that the lower the exercise level, the more likely bus drivers are to experience stress peaks.^19^ While drivers in Chapecó are physically active, they also experience a moderate level of stress, differently from what has been observed in other regions.

We had limitations during the course of the study. One of these was the pandemic, during which it was not possible to collect the answers via a printed form due to the guidelines for fighting against COVID-19, which required the answers to be collected online. Also, some employees showed no interest in answering the questionnaire, some claimed they had difficulty to understand the questionnaire because it was online and some claimed they had no time to fill in the form because of their short breaks. Furthermore, it is possible that respondents who chose not to complete the questionnaire had worse health conditions and exercised less. However, this does not mean that the sample is unrepresentative of the population of bus drivers in Chapecó, nor does it exclude judiciousness in the choice of assessment instruments, their application, and analysis.

Further studies should be conducted to explore aspects aimed at health promotion for these workers, encouraging changes in their habits and behavior, and introducing exercise programs to improve the quality of life of bus drivers.
